# Point-of-care ultrasound can make the difference in patients with heart failure at primary care

**DOI:** 10.1093/fampra/cmaf068

**Published:** 2025-08-26

**Authors:** Jonathan dos Santos, José Ribeiro, Francisco R Gonçalves, Alexandra Gonçalves

**Affiliations:** Faculty of Medicine, University of Porto, Porto 4200-319, Portugal; CINTESIS, Center for Health Technology and Services Research, Porto 4200-450, Portugal; Family Medicine Department, Hospital Lusíadas Paços de Ferreira, Paços de Ferreira 4590-073, Portugal; Department of Medical Sciences, University of Aveiro, Aveiro 3810-193, Portugal; Cardiology Department, Centro Hospitalar Vila Nova de Gaia/Espinho, Vila Nova de Gaia 4434-502, Portugal; Faculty of Medicine, University of Porto, Porto 4200-319, Portugal; Faculty of Medicine, University of Porto, Porto 4200-319, Portugal

**Keywords:** family medicine, primary healthcare, heart failure, point-of-care ultrasound, echocardiography, clinical decision-making, risk stratification

## Abstract

**Background:**

Primary healthcare centers (PHC) play a pivotal role in the first-line management of patients with diabetes and hypertension, major risk factors for heart failure (HF) development. Point-of-care cardiac ultrasound (POCUS), integrated as an extension of the physical examination, holds significant potential to enhance diagnostic accuracy and clinical management in this setting.

**Objectives:**

Evaluate the impact of POCUS on clinical decision-making in patients with HF and at risk of developing HF in PHC and compare POCUS findings with clinical assessment alone, conventional echocardiography, and electrocardiogram results.

**Methods:**

Patients with diabetes, hypertension, or HF symptoms at a PHC underwent POCUS by a trained family physician. The findings were compared with traditional clinical practice. Decisions regarding referral for an echocardiogram or hospital consultation were contrasted with those of two clinicians who do not use POCUS, and the investigator's echocardiographic results were compared with those from conventional echocardiography and electrocardiogram. Data were analyzed using SPSS.

**Results:**

Among 196 patients (66 ± 15 years; 53.6% female), 36.2% had HF symptoms, 89.2% hypertension, and 29.7% diabetes. Investigator requested less echocardiograms (44 vs. 145 and 125) and made less hospital referral (15 vs. 16 and 24). Using POCUS, congestive patients were less than expected (18 vs. 43 cases), and stage B HF patients were more than clinically (44.9% vs. 19.4%). POCUS identified more cases with left ventricular hypertrophy than electrocardiograms (58 vs. 10).

**Conclusion:**

These findings highlight the value of integrating POCUS into routine family physician consultations, particularly for the management of HF and effective risk stratification.

## Introduction

Heart failure (HF) remains a major contributor to the global burden of cardiovascular disease, driven by increased life expectancy and a corresponding rise in its prevalence, mortality, and hospitalization rates, particularly among older populations. HF significantly impacts patients’ quality of life and imposes considerable strain on healthcare systems worldwide. However, this economic and clinical burden can be mitigated through early identification and timely intervention, including the optimization of both pharmacological and non-pharmacological strategies [1–3].

According to the American Heart Association guidelines, HF is categorized into four stages [4]. Stages A and B, collectively termed “preclinical HF,” include patients who do not exhibit clinical symptoms [5]. Stage A (at-risk for HF) encompasses individuals at risk of developing HF due to predisposing conditions but without evidence of structural or functional cardiac abnormalities. In contrast, Stage B (pre-HF) refers to patients with structural or functional cardiac abnormalities that remain asymptomatic and undetectable on physical examination [6]. Echocardiography serves as a critical diagnostic tool for identifying these abnormalities, highlighting the necessity of effective comorbidity management to improve prognostic outcomes in HF [4–8].

Accurate identification of HF in its early stages is a crucial first step in effective management [5, 8, 9]. Family physicians (FP) are uniquely positioned to play a pivotal role in this process, as they frequently oversee the care of patients with HF and those at elevated risk for its development, including individuals with diabetes, arterial hypertension, atrial fibrillation, or coronary artery disease [10, 11]. However, disparities in medical and diagnostic resources across primary healthcare setting pose challenges, particularly in rural or underserved areas [12, 13].

The electrocardiogram (ECG), a standard diagnostic tool in primary healthcare centers, has limited sensitivity and specificity for detecting HF, especially in its early stages [14]. Although natriuretic peptides are an effective screening tool, their diagnostic accuracy is higher in patients presenting with symptoms of HF and those with systolic dysfunction than in asymptomatic patients or with diastolic dysfunction [15].

Echocardiography, particularly through the integration of point-of-care ultrasound (POCUS) in primary healthcare settings, performed by trained FP, could mitigate these limitations by facilitating the early detection of structural abnormalities in Stage B HF. Moreover, POCUS has the potential to enhance the diagnostic capacity of physical examinations in patients with Stage A HF, offering a cost-effective and practical solution, especially in resource-constrained or rural healthcare settings [14–19]. Additionally, patients with HF frequently present with symptoms or signs of congestion, necessitating distinct treatment approaches based on the type of congestion: intravascular or interstitial. Differentiating between these forms is essential for tailoring therapeutic strategies effectively. POCUS plays a crucial part in this context, particularly by enabling the assessment of the inferior vena cava (IVC), a parameter that reflects left atrial pressure. Accurate evaluation of the IVC via POCUS aids in guiding diuretic therapy, thereby optimizing the management of intravascular congestion and the improving patient outcomes [20].

This study aimed to evaluate the utility of POCUS, performed by a trained FP, in the classification of HF patients and its role in guiding diagnostic and therapeutic decision-making in primary healthcare settings.

## Methods

### Standard protocol approvals, registrations, and patient consents

The study was approved by the Ethics Committee of the Northern Regional Health Administration (ARS Norte) under reference number 50/2018. All procedures were conducted following the ICH Harmonized Tripartite Guidelines for Good Clinical Practice and with the Declaration of Helsinki. All participants provided written informed consent prior to inclusion in the study.

### Study design and population

Prospective observational study, conducted between 2021 and 2022, at a rural primary healthcare center. The study population comprised patients under the care of the FP, who also served as the study investigator, during both office consultations and home visits.

**Figure 1.**
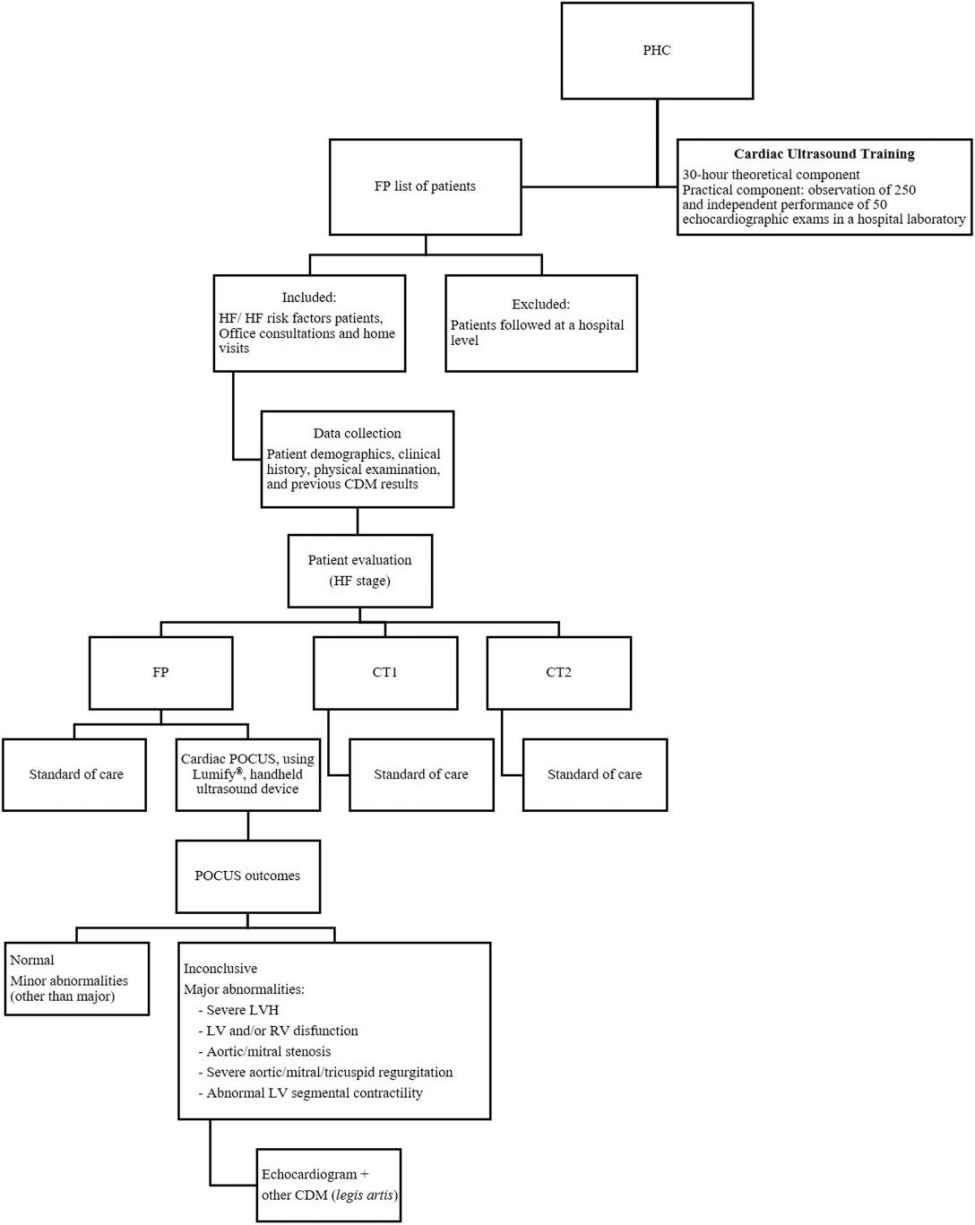
Study design and methodology flowchart. CDM, complementary diagnostic methods; CT1 and CT2, independent control family physicians; FP, family physician, the study investigator; HF, heart failure; LV, left ventricle; LVH, left ventricular hypertrophy; PHC, primary healthcare center; POCUS, point-of-care ultrasound; RV, right ventricle.

Within the study period, all patients with presence of HF signs or symptoms, or HF risk factors, including diabetes and hypertension (Fig. 1) with indication to perform an echocardiogram were eligible to participate in the study. Patients receiving follow-up care at a hospital level were excluded.

Eligible patients were identified by the investigator during clinical practice and underwent POCUS at the same consultation. Prior to inclusion, patients had time to ask any question about the study procedures and gave their written informed consent to participate. In fewer than 10% of cases, POCUS was scheduled for a follow-up consultation due to time constraints.

### Training and equipment

A single investigator (the FP who also served as the study investigator) performed POCUS as a complement to the patients’ medical history and physical examination. Prior to conducting the study, this investigator completed structured cardiac ultrasound training, which included a theoretical component (certified 30-hour online course) and a practical component (observation of 250 echocardiographic exams and independent performance of 50 exams in a hospital echocardiography laboratory).

POCUS assessments were performed using a handheld ultrasound device (Lumify**®**).

### Clinical and ultrasound data collection

Data collected included patient demographics (age, sex), reason for consultation, clinical signs and symptoms, and physical examination findings. Clinical history, and ECG and echocardiograms’ prior results were obtained from patient medical records.

POCUS views obtained included: parasternal long axis, parasternal short axis, apical four-chamber, apical three-chamber, apical two-chamber, and subcostal views. Color Doppler imaging was used to assess valvular disease, and the following measurements were recorded: ventricular wall thickness (posterior and septal wall), left ventricular end-diastolic diameter, left atrial diameter, ascending aorta diameter, and IVC diameter. The presence of pleural and pericardial effusions was also evaluated. Ventricular function was qualitatively assessed by visual estimation (“eyeballing”).

A conventional echocardiogram was asked to be performed a maximum of one month after the POCUS.

### POCUS interpretation

POCUS findings were categorized into four outcomes: inconclusive, no alterations, minor alterations, and major alterations (Fig. 1). HF staging was determined according to the American College of Cardiology/American Heart Association (ACC/AHA) stages A to D [4]. Volume status was assessed based on IVC diameter and contractility.

Clinical management decisions were made by the FP who also served as the study investigator based on the patient's medical history, physical examination, and POCUS findings, particularly in cases of inconclusive or significant alterations. These decisions included therapeutic adjustments, patient education, hospital referrals, and requests for conventional echocardiography or additional diagnostic tests.

### Control physician evaluation

Clinical information, namely clinical history, laboratory tests, ECG and echocardiograms’ prior results, was shared with two control FP (CT1 and CT2) who, based on that information, independently decided whether to request an echocardiogram or refer the patient to the hospital (routine consultation or emergency department). These physicians had no access to POCUS data.

### Statistical analysis

Descriptive and analytical statistical analyses were performed using SPSS software. Categorical variables were summarized as absolute and relative frequencies, while continuous variables were expressed as mean ± standard deviation (SD) or median with percentiles.

Decisions and outcomes based on POCUS were compared with CT1 and CT2 decisions and with conventional methods. For each observer and defined outcome, the proportions of overall and partial agreement were calculated, with respective Cohen's Kappa. A significance level of α = 0.05 was used in all analyses.

No imputation of missing or incomplete values was performed.

## Results

### Patient characteristics

The study included a convenience, consecutive sample of 196 patients, 53.6% female, mean age 66 years old (range, 23–95). Patient characteristics are summarized in Table 1. The primary reason for consultation was routine follow-up (92.3%), with a minority occurring in acute contexts (7.7%). Most evaluations were performed in the medical office (96.4%), while seven patients were assessed during home visits. Among the study population, 63.8% were asymptomatic, 89.2% had arterial hypertension and 29.7% diabetes.

**Table 1.** Patient characteristics.

	*n* = 196
Age in years, mean (±SD)	66.1 (±14.5)
Female sex, *n* (%)	105 (53.6)
BMI, kg/m^2^, mean (±SD)	29.1 (±5.2)
Consultation, *n* (%)	
Routine/follow-up consultation	181 (92.3)
Acute consultation	15 (7.7)
Signs/symptoms, *n* (%)	
Symptomatic	71 (36.2)
Ankle swelling	32 (16.3)
Bendopnea	15 (7.7)
Dyspnea	40 (20.4)
Elevated jugular venous pressure	16 (8.2)
Fatigue	62 (31.6)
Heart murmur	37 (18.9)
Orthopnea	15 (7.7)
Pulmonary crepitations	17 (8.7)
Reduced exercise tolerance	68 (34.7)
NYHA class, *n* (%)	
NYHA I	112 (57.1)
NYHA II	57 (29.1)
NYHA III	23 (11.7)
NYHA IV	2 (1.0)
UNK	2 (1.0)
Comorbidities, *n* (%)	
Arrhythmia	17 (8.7)
Atrial fibrillation	22 (11.3)
Coronary artery disease	2 (1)
Diabetes	58 (29.7)
Hypertension	174 (89.2)

BMI, body mass index; *n*, number of patients; NYHA, New York Heart Association HF functional classification; SD, standard deviation; UNK, unknown.

### POCUS impact on echocardiography requests and hospital referrals

Among 194 patients, the investigator requested fewer echocardiograms (*n* = 44) compared to the two independent FP who did not use POCUS, CT1 (*n* = 142) and CT2 (*n* = 125; Table 2). The global level of agreement between the investigator and CT1 was low (*k* = 0.109), and no kappa calculation was able to be performed with CT2. The agreement rates between the two control physicians were higher for cases where no echocardiogram was requested (68%) compared to cases where an echocardiogram was requested (43%), although with a *k* = 0.099.

**Table 2.** Proportion of agreement between investigator and control physicians on echocardiography requests and hospital referrals.

Echocardiography requests							
n = 193		Investigator (n)		Proportion of agreement			Kappa
		No	Yes	Global	No request	Request	
CT1	No	46	5	44% [37%–51%]	90% [79%–96%]	27% [21%–35%]	0.109 [0–0.211]
	Yes	103	39				

*n* = 194		Investigator (*n*)		Proportion of agreement			Kappa
		No	Yes	Global	No request	Request	
CT2	No	51	18	40% [33%–47%]	73% [62%–83%]	21% [15%–29%]	-
	Yes	99	26				

n = 194		CT2 (*n*)		Proportion of agreement			Kappa
		No	Yes	Global	No request	Request	
CT1	No	22	29	61% [54%–68%]	43% [31%–57%]	68% [60%–75%]	0.099 [0–0.258]
	Yes	46	97				

Hospital referrals							
*n* = 194		Investigator (*n*)		Proportion of agreement			Kappa
		No	Yes	Global	No referral	Referral	
CT1	No	169	9	90%	95%	38%	0.334 [0.049–0.618]
	Yes	10	6	[85%–94%]	[91%–97%]	[19%–61%]	

*n* = 195		Investigator (*n*)		Proportion of agreement			Kappa
		No	Yes	Global	No referral	Referral	
CT2	No	159	12	83%	93%	13%	0.065 [0–0.356]
	Yes	21	3	[77%–88%]	[88%–96%]	[4%–31%]	

CT1, Control family physician 1; CT2, Control family physician 2; *n*, number of patients.

Considering the hospital referral decisions for consultation or emergency services (Table 2), the investigator referred fewer patients (*n* = 15) compared to CT1 (*n* = 16) and CT2 (*n* = 24). Agreement rates for referred cases were lower (38% for CT1 and 13% for CT2) compared to non-referred cases (95% and 93%, for CT1 and CT2, respectively).

### Assessing cardiac abnormalities: POCUS vs. Conventional Echocardiography

The efficacy of POCUS was compared with a conventional echocardiogram by evaluating the presence or absence of cardiac abnormalities. The conventional echocardiogram was performed at a median of 27 ± 31 days after the POCUS. The overall concordance rate was 83%, with partial concordance of 70% for cases without abnormalities and 92% for cases with abnormalities. When findings were stratified into “no abnormalities,” “minor abnormalities,” and “major abnormalities”; the overall concordance was 64%. Partial concordance rates were 30%, 67%, and 82% for “no abnormalities,” “minor abnormalities,” and “major abnormalities,” respectively (Table 3).

**Table 3.** Proportion of agreement between POCUS and conventional echocardiography in detecting cardiac abnormalities.

*n* = 72		POCUS (*n*)		Proportion of agreement			Kappa
		No	Yes	Global	No	Yes	
Conventional Echocardiography	No	3	7	83% [73%–90]	70% [40%–89%]	92% [82%–97%]	0.200 [0–0.632]
	Yes	5	57				

*n* = 72		POCUS (*n*)			Proportion of agreement				Kappa
		No	Minor^a^	Major^b^	Global	No	Minor^a^	Major^b^	
Conventional Echocardiography	No	3	7	0	64% [52%–74%]	30% [11%–60%]	67% [53%–78%]	82% [52%–95%]	0.302 [0.088–0.517]
	Minor^a^	8	34	9					
	Major^b^	0	2	9					

BSA, body surface area; LV, left ventricle; LVH, left ventricular hypertrophy; *n*, number of patients; POCUS, point-of-care ultrasound; RV, right ventricle.

^a^Any abnormality other than major.

^b^Severe LVH—indexed LV mass/BSA ≥122 g/m^2^ (women) and ≥149 g/m^2^ (man), LV and/or RV disfunction (eyeball), aortic/mitral stenosis (B mode and color mode evaluation), severe aortic/mitral/tricuspid regurgitation (B mode and color mode evaluation), abnormal LV segmental contractility.

### Volume status assessment: physical examination alone vs. physical examination with POCUS

Patient volume status was accessed using both physical examination and POCUS. POCUS evaluation included measurement of IVC diameter, considered elevated if >20 mm (Supplementary Table S1). Concordance between physical examination and physical examination with POCUS was higher in cases without congestion (82%) compared with those with congestion (72%).

### HF staging: conventional method vs. POCUS

HF stages (A–D) were assessed during the same consultation using clinical history, including prior imaging results, and physical examination, followed by the addition of POCUS data. POCUS significantly influenced the classification of HF stages, particularly in identifying stage B patients (44.9% vs. 19.4% with conventional method). Conversely, the conventional method identified more cases at stage A (43.9% vs. 17.9% with POCUS). Concordance rates between methods varied substantially: 12% for stage A, 87% for stage B, and 97% for stage C (Table 4).

**Table 4.** Proportion of agreement in HF staging between POCUS and conventional consultation.

*n* = 196		With POCUS (*n*)				Proportion of agreement				Kappa
		A	B	C	D	Global	A	B	C	
Without POCUS	A	30	55	1	0	68% [61%–74]	12% [6%–20%]	87% [73%–94%]	97% [90%–97%]	0.542 [0.449–0.635]
	B	5	33	0	0					
	C	0	0	70	2					
	D	0	0	0	0					

*n*, number of patients; POCUS, point-of-care ultrasound.

### Assessing left ventricular hypertrophy: ECG vs. POCUS

The ability of POCUS and ECG to detect left ventricular hypertrophy (LVH) was also compared (Supplementary Table S2). POCUS identified 58 cases of LVH, whereas ECG indicated 10. The overall agreement between the two methods was 65%, with 50% concordance for cases with LVH and 66% for cases without LVH.

## Discussion

### Role of cardiac POCUS in family practice

This observational study highlights the transformative potential of cardiac POCUS as a diagnostic tool in family practice consultations. By augmenting traditional clinical evaluation methods, POCUS enhances diagnostic accuracy and decision-making, particularly in detecting subclinical cardiac abnormalities that might be missed by physical examination and electrocardiography. These findings align with prior research demonstrating the effectiveness of POCUS, especially when integrated with remote expert support systems [21, 22].

POCUS is especially valuable for identifying clinically significant conditions, such as LVH and early stages of HF, and for distinguishing serious pathologies from minor abnormalities [22, 23]. However, the technical skills required for cardiac ultrasound and the time investment may hinder broader adoption, challenges that advancements in artificial intelligence (AI) could mitigate by improving image acquisition and interpretation efficiency [24].

POCUS integration in primary care has been increasingly studied. However, most of the existing research focuses on undergraduate training programs, its use in emergency settings, or feasibility and accuracy studies with remote supervision or support. To the best of our knowledge, only one study was performed in the outpatient setting, where FP performed cardiac POCUS, although specialists conducted image interpretation remotely [21]. In contrast, our study evaluates the real-life implementation of cardiac POCUS in routine family practice consultations, where the FP acted autonomously as operator, interpreter, and clinical decision-maker without external support. This reflects the practical reality of primary care in rural settings, such as in Portugal, and highlights the potential for immediate integration of POCUS into everyday clinical workflows to improve patient care.

### Impact on resource utilization

POCUS significantly reduces the number of echocardiograms requested and hospital referrals, contributing to improved healthcare efficiency. The investigator requested fewer echocardiograms and made fewer hospital referrals than the control physicians, indicating POCUS's utility in tailoring on-site management. This reduction aligns with studies demonstrating that POCUS minimizes unnecessary advanced imaging and streamlines diagnostic pathways [25–27].

Despite its benefits, variability in referral decisions among control physicians underscores the need for standardized echocardiogram referral guidelines to ensure consistency and reliability across primary care practices.

### HF staging and LVH detection

POCUS enhances the classification of HF stages, with a notable increase in stage B HF diagnoses compared to conventional methods (44.9% vs. 19.4%). This improvement is critical, as early identification of stage B HF allows for timely preventive measures, such as lifestyle modifications and optimized pharmacological treatments, potentially delaying progression to symptomatic stages C and D [23].

Although the increased number of Stage B HF diagnoses with POCUS should be interpreted with caution in the absence of long-term outcome data, it is important to highlight that both Stage A and B HF patients are asymptomatic, with Stage B being characterized by structural or functional cardiac abnormalities that would otherwise remain undetected without imaging. In primary care, Stages A and B are expected to be the most frequent, but differentiating between them is challenging without echocardiography.

Our findings demonstrate that POCUS, as an extension of the physical examination, allows the detection of such abnormalities, leading to reclassification from Stage A to B. While long-term data are needed to assess outcome implications, identifying Stage B HF patients enables earlier targeted interventions and closer monitoring, which may prevent progression to symptomatic stages. This raises an important question regarding whether current follow-up strategies in primary care should be revisited to integrate tools like POCUS for improved risk stratification and disease prevention.

Additionally, POCUS demonstrated superior sensitivity for detecting LVH, identifying 58 cases compared to only 10 detected by ECG. This discrepancy emphasizes ECG's limitations, particularly in elderly patients with comorbidities [28, 29]. LVH detection is crucial for assessing cardiovascular risk and informing interventions, such as antihypertensive therapy adjustments or closer cardiac monitoring [28–30].

### Congestion status evaluation

The ability to evaluate congestion status is integral to HF management. POCUS complements physical examination by assessing IVC diameter, allowing differentiation between intravascular and interstitial congestion. This distinction guides targeted therapeutic strategies [20, 31]. However, it is important to acknowledge the limitations of relying solely on IVC assessment. Factors such as chronic dilation, tricuspid regurgitation, or variations due to physical conditioning (e.g. young athletes) can influence IVC measurements, leading to potential misinterpretation [32].

To overcome these challenges, integrating advanced protocols, such as the Venous Excess Ultrasound (VExUS) score and pulmonary B-line analysis, could provide more comprehensive assessments. These tools have been shown to enhance the accuracy of congestion evaluation, offering a more robust framework for therapeutic decision-making [33, 34].

### Enhancing clinician–patient engagement

From both the clinician's and patient's perspectives, the integration of POCUS enhances awareness of disease progression. Providing visual evidence of cardiac abnormalities during consultations may encourage greater adherence to preventive strategies and therapeutic regimens. Additionally, educating patients on imaging findings reinforces the importance of treatment adherence, which has been shown to improve clinical outcomes [30].

The ability to visually demonstrate abnormalities during consultations fosters patient trust and engagement, enabling a more collaborative approach to managing chronic conditions. This aspect of POCUS can help improving outcomes by applying guideline medical directed therapy and also contribute to a more patient-centered healthcare model. Future longitudinal studies are needed to confirm any direct benefits on patient outcomes.

### Challenges and future directions

Despite its considerable potential, this study has several limitations. The patient cohort, while representative of a typical FP list, is relatively small and may not reflect the diversity of the entire Portuguese population. Nevertheless, the predominantly elderly demographic provides valuable insights into a population segment where the diagnosis of subclinical disease is becoming increasingly important across Europe [3, 15]. This focus on older patients aligns with the demographic shift in many European countries, where an aging population emphasizes the need for improved detection and management of chronic conditions [3, 4].

Another challenge is the variability in ultrasonographic proficiency among FP. While the investigator demonstrated expertise in POCUS, these skills may not be generalizable to all practitioners, underscoring the need for standardized training programs and continuous professional development [18, 21]. However, evidence suggests that structured training programs, even of short duration, can significantly improve diagnostic performance. Kobal *et al*.[35] showed that first-year medical students, after only 18 hours of echocardiography training, were able to identify 75% of cardiac pathologies with handheld ultrasound, outperforming board-certified cardiologists relying on physical examination alone.

Additionally, the POCUS single-operator model might have introduced interpretation and selection bias. The investigator had all the relevant clinical information about the patients as their FP but also a significant level of confidence on POCUS data as it sole operator.

Factors such as geographic location (urban versus rural), availability of diagnostic resources, and variations in clinician skills must also be considered to determine the generalizability and scalability of these results. Future research should focus on validating these findings across diverse populations and healthcare settings, particularly in resource-limited environments. Additionally, incorporating AI into POCUS workflows could enhance efficiency and accuracy, further expanding its utility in routine care. Longitudinal studies evaluating the cost-effectiveness and long-term outcomes of POCUS integration are also warranted [24, 33].

## Conclusions

Cardiac POCUS represents a significant advancement in family practice, offering enhanced diagnostic accuracy and facilitating more informed clinical decision-making. It improves the detection of cardiac abnormalities, including LVH and subclinical HF stages, and contributes to early, targeted interventions that can delay disease progression and improve patient outcomes.

The integration of POCUS into routine consultations reduces unnecessary echocardiogram requests and hospital referrals, optimizing healthcare resource utilization. Furthermore, its ability to provide visual diagnostic insights during consultations fosters patient engagement and adherence to care plans, leading to better health outcomes.

However, the study's limitations, including the small, localized sample size and variability in operator expertise, highlight the need for broader validation. Standardized training and advanced diagnostic protocols are essential to ensure consistent application. Future efforts should also explore the integration of AI technologies to address existing challenges and maximize the utility of POCUS in primary care.

POCUS has the potential to revolutionize primary care by offering a comprehensive, immediate, and patient-centered approach to cardiovascular assessment. Its widespread adoption, supported by research and innovation, will pave the way for more effective and efficient management of cardiovascular conditions in family practice.

## Supplementary Material

cmaf068_Supplementary_Data

## Data Availability

Anonymized data are available by request from the corresponding author on reasonable request.
