# Supplementary material for: Point-of-care ultrasound can make the difference in patients with heart failure at primary care
Source: Fam Pract. 2025 Aug 26;42(5):cmaf068. doi: 10.1093/fampra/cmaf068 (PMC12411906; doi:10.1093/fampra/cmaf068)
Supplement: cmaf068_Supplementary_Data [file cmaf068_supplementary_data.pdf]

Supplementary Table 1. Proportion of agreement of hypervolemia status between physical examination with and without POCUS.

n= 189		With POCUS (n)		Proportion of agreement			Kappa
		No	Yes	Global	No	Yes	
Without POCUS	No	141	5	81%	82%	72%	0.337
	Yes	30	13	[75%-86]	[76%-87%]	[49%-88%]	[0.139-0.534]

Legend: Using POCUS as an extended physical examination, hypervolemia status was reviewed according to IVC diameter (>20mm). IVC – inferior vena cava; n, number of patients; POCUS – point-of-care ultrasound.

Supplementary Table 2. Proportion of agreement between POCUS and ECG in LVH detection.

n= 168		POCUS (n)		Proportion of agreement			Kappa
		No	Yes	Global	No	Yes	
ECG	No	105	53	65%	66%	50%	0.051
	Yes	5	5	[58%-73]	[59%-73%]	[24%-76%]	[0.0-0.248]

Legend: Screening LVH using ECG report was followed by POCUS evaluation according to indexed LV mass/BSA (≥96 g/m^2 for women and ≥108 g/m^2 for man). On the left side are absolute values and on the right side the results of the proportion of agreement. BSA – body surface area; LV – left ventricle; LVH – left ventricular hypertrophy; n, number of patients; POCUS – point-of-care ultrasound.
